# Publisher Correction: Observational social learning of “know-how” and “know-what” in wild orangutans: evidence from nest-building skill acquisition

**DOI:** 10.1038/s42003-025-08665-w

**Published:** 2025-08-28

**Authors:** Andrea L. Permana, Junaidi Jaka Permana, Lara Nellissen, Eggi Septian Prayogi, Didik Prasetyo, Serge A. Wich, Carel P. van Schaik, Caroline Schuppli

**Affiliations:** 1 Ape Tank, Department of Psychology, University of Warwick, Coventry, UK; 2 Development and Evolution of Cognition Research Group, Max Planck Institute of Animal Behaviour, Konstanz, Germany; 3 Eco-Anthropologie, Muséum National d′Histoire Naturelle, Paris, France; 4 Department of Biology, Faculty of Biology and Agriculture, Universitas Nasional, Jakarta, Indonesia; 5 School of Biological and Environmental Sciences, Liverpool John Moores University, Liverpool, UK; 6 Institute for Biodiversity and Ecosystem Dynamics, University of Amsterdam, Amsterdam, The Netherlands; 7 Department of Evolutionary Anthropology, University of Zurich, Zurich, Switzerland; 8 Department of Ecology and Animal Societies, Max Planck Institute for Animal Behaviour, Konstanz, Germany; 9 Institute for the Interdisciplinary Study of Language Evolution, University of Zurich, Zurich, Switzerland

**Keywords:** Behavioural ecology, Attention

Correction to: *Communications Biology* 10.1038/s42003-025-08217-2, published online 7 June 2025

In the version of the article initially published, three references were missing that should have been cited in the sixth and seventh paragraphs of the Introduction (Whiten, A. 10.1016/j.plrev.2022.10.003 (2022); Tennie, C. et al. 10.1007/s10539-020-09769-9 (2020); and Tennie, C. The earliest tools and cultures of hominins. in *Oxford Handbook of Cultural Evolution* (Oxford University Press, 2023). The references have been inserted in the HTML and PDF versions of the article.

