# Supplementary material for: Observational social learning of “know-how” and “know-what” in wild orangutans: evidence from nest-building skill acquisition
Source: Commun Biol. 2025 Jun 7;8:890. doi: 10.1038/s42003-025-08217-2 (PMC12145437; doi:10.1038/s42003-025-08217-2)
Supplement: Supplementary file 1 — Supplementary Material [file 42003_2025_8217_MOESM1_ESM.pdf]

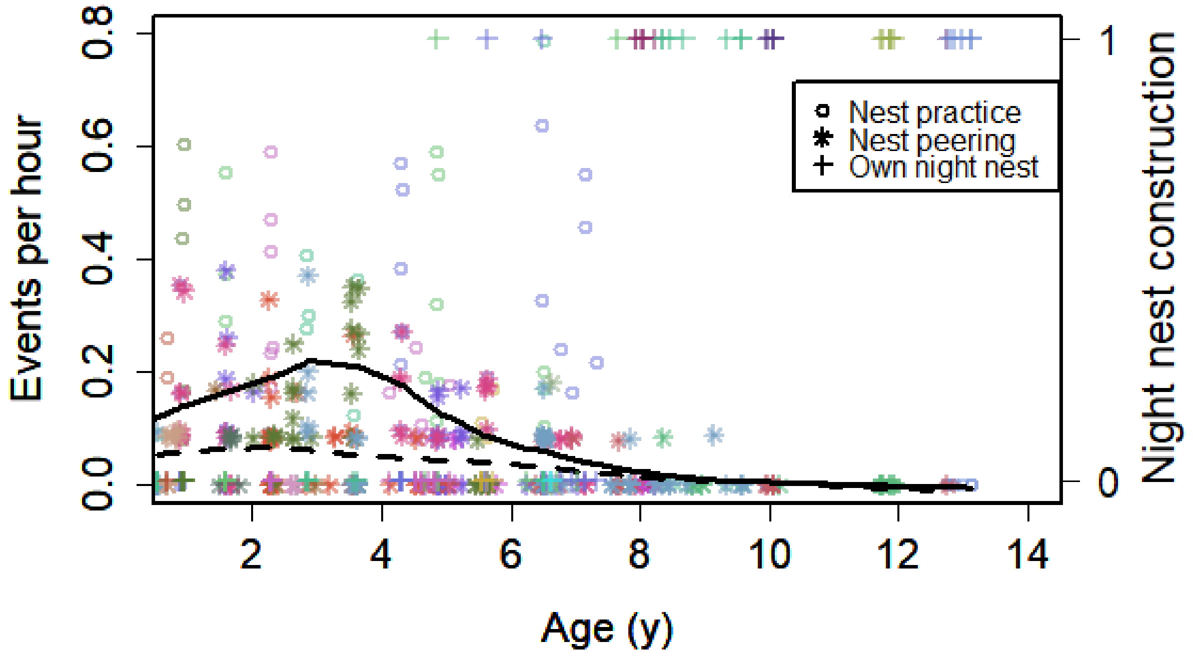

Supplementary Figure 1: Age trajectories for nest peering, nest practice and the use of the own night nest in immatures. Daily nest peering rates, daily nest practice rates and successful construction of a night nest in relation to the age (years) of the immature focal individuals during full day follows (supplemented from [7,24]). For night nest construction, 1 means that the individual built and slept in their own night nest on that day and 0 means that the individual did not build a night nest on that day (or attempted to but failed). The different colours represent the different focal individuals that contributed data to this analysis (n = 20). The solid line represents the regression line for nest practice and the dashed line the regression line for nest peering. Both regression lines were fitted using the loess function.

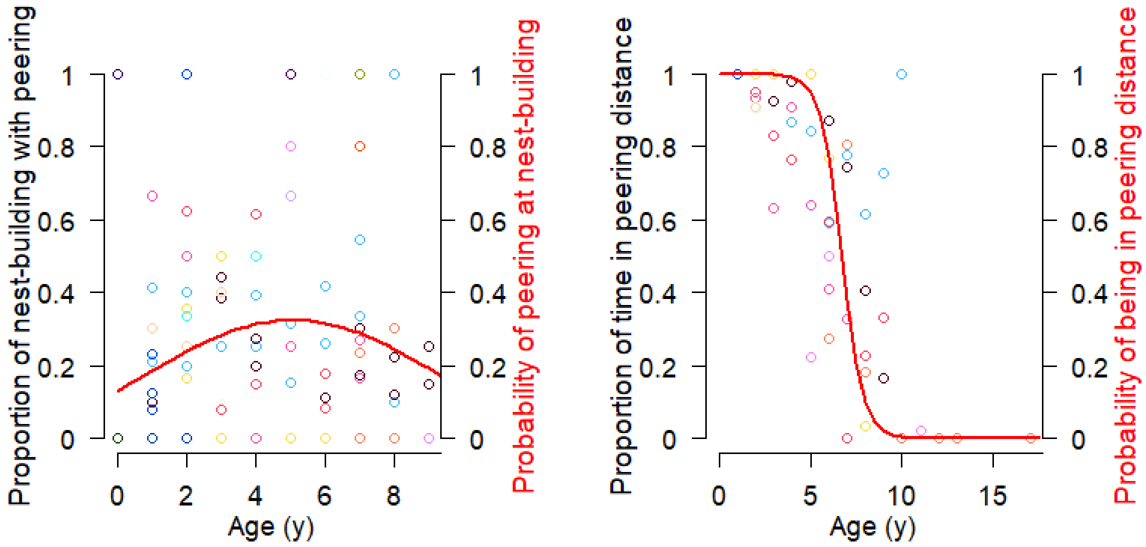

Supplementary Figure 2: Probability of peering at the mother's nest and probability of being within peering distance when the mother made a nest. a) The proportion of nest-building events of mothers during which their infants peered (calculated per individual and yearly age class) and the predicted probability that immatures peer at their mothers' nest-building (based on Model II). **b)** The proportion of scans immatures spent within peering distance (i.e., within 5 meters) of the mother when the mother was building a nest and the predicted probability of immatures being within peering distance during the mother's nest-building events (based on the model shown in Supplementary Table 4). The different colours represent the different focal individuals that contributed data to this analysis (a: *n* = 27, b: *n*= 10).

Supplementary Figure 3. Jaccard Similarity Index. The Jaccard similarity index was used to compare the percentage overlap of nest tree species used between dyad pairs. The resulting index measures the similarity between two sample sets.

$$\text{Jaccard similarity index} = \frac{\text{Number of species shared by both sets}}{\text{Total number of species in both sets}} \times 100$$

Supplementary Table 1. Summary of focal individuals. Names, age class, sex, known relationships, the number of their nests included in the species selectivity analyses, and to which peering/practice analyses they contributed data.

Focal	Sex	Age class	Mother's name	No. Nests included in species analysis	Contributed data to models
Albin	M	Dependent	Alice	NA	II
Alice	F	Mother	Unknown	18	I, IV
Amor	M	Dependent	Alice	NA	II, III
Benito	M	Dependent	Benita	NA	II
Cheech	M	Dependent	Chick	NA	II, III
Chindy	F	Dependent	Cissy	22	II, III, IV
		Independent		14	IV
Chuck	M	Dependent	Chick	NA	II
Cinnamon	F	Dependent	Cissy	21	II, III
		Independent		6	IV
Cissy	F	Mother	Unknown	152	I, IV
Dalia	F	Dependent	Dodi	NA	II
Diddy	M	Dependent	Dodi	NA	II, III
		Independent		12	IV
Dodi	F	Mother	Unknown	13	IV
Eden	F	Dependent	Ellie	32	II, III, IV
Ellie	F	Independent	Friska	33	II, III, IV
	F	Mother		322	IV
Frankie	M	Dependent	Friska	23	II, III, IV
Fredy	M	Dependent	Friska	35	II, III, IV
		Independent		22	IV
Friska	F	Mother	Unknown	301	I, IV
Goli	M	Dependent	Gani	NA	II
Gura	M	Independent	Unknown	NA	III
Inky	M	Dependent	Infant	NA	II, III

Karma	F	Mother	Unknown	13	IV
Kronos	M	Dependent	Karma	NA	II
Lilly	F	Dependent	Lisa	33	II, III, IV
		Independent		41	IV
		Mother		29	I, IV
Lisa	F	Mother	Cissy	299	I, IV
Lois	M	Dependent	Lisa	51	II, III, IV
		Independent		51	IV
Luther	M	Dependent	Lisa	NA	II, III
Nora	F	Mother	Unknown	NA	I
Nuk	M	Dependent	Nora	2	II
Olala	F	Dependent	Okume	NA	II, III
Pepito	M	Dependent	Piniata	NA	II, III
Piniata	F	Mother	Unknown	4	I
Raffi	F	Mother	Unknown	49	I, IV
Rendang	M	Dependent	Raffi	NA	II, III
Ronaldo	M	Dependent	Raffi	NA	II, III
Sarabi	F	Mother	Unknown	31	I, IV
Sazu	M	Dependent	Sarabi	NA	II, III
		Independent		6	IV
Shera	F	Independent	Chick	NA	III
Simba	M	Dependent	Sarabi	NA	II, III, IV
Sound	M	Dependent	Sonya	NA	II
Tiara	F	Mother	Unknown	13	I, IV
Tina	F	Independent	Rafi	40	III, IV
Tornado	M	Independent	Tiara	NA	II, III
Trident	M	Independent	Tiara	26	II, III, IV
Yulia	F	Independent	Unknown	NA	III

Supplementary Table 2. Day nest tree species use. Use of day nest tree species according to age class; (a) dependent immatures, (b) independent immatures and (c) mothers.

(a)

**Dependent immatures**									
**Day Nest Tree Species**	**Family**	**Chindy**	**Cinnamon**	**Eden**	**Frankie**	**Fredy**	**Lilly**	**Lois**	**Total**
Jackiopsis ornata	Rubiaceae	1	3		1	2		1	**8**
Syzygium densiflora	Myrtaceae		3	5	1			10	**19**
Xylopia malayana	Annonaceae		1			1		1	**3**
Garcinia sp.	Clusiaceae	1						1	**2**
Litsea gracilipes	Lauraceae		1	4	1	1	1	2	**10**
Baccaurea sp.	Euphorbiaceae			1					**1**
Campnosperma auriculatum	Anacardiaceae			1					**1**
Tetramerista glabra	Tetrameristaceae	1	1	2	2	3	5	12	**26**
Mangifera gracilipes	Anacardiaceae							2	**2**
Shorea teysmanniana	Dipterocarpaceae				2	1		2	**5**
Gymnacranthera contracta	Myristicaceae			1	1				**2**
Sandoricum beccarianum	Meliaceae	3	1	1	1	6	7		**19**
Gluta renghas	Anacardiaceae	2	1	8	5	5	2		**23**
Parastemon urophyllus	Chrysobalanaceae	1	1	1		9	1	5	**18**
Dialium patens	Fabaceae				1	3		2	**6**
Brackenridgea palustris	Ochnaceae		1						**1**
Sterculia oblongata	Sterculiaceae	1	3	2		1		2	**9**
Garcinia havilandii	Clusiaceae		2						**2**
Horsfieldia polyspherula	Myristicaceae	5	2	5	2	2	2	5	**23**
**Total**		**15**	**20**	**31**	**17**	**34**	**18**	**45**	**180**

(b)

**Independent Immatures**										
**Day Nest Tree Species**	**Family**	**Chindy**	**Diddy**	**Ellie**	**Fredy**	**Lilly**	**Lois**	**Sazu**	**Trident**	**Total**
Dillenia pulchella	Dilleniaceae						1			**1**
Alstonia pneumatophora	Apocynaceae					1				**1**
Jackiopsis ornata	Rubiaceae					2	1			**3**
Syzygium densiflora	Myrtaceae	1	1		1	1	1	1	1	**7**
Xylopia malayana	Annonaceae					1				**1**
Garcinia sp.	Clusiaceae			3						**3**
Litsea gracilipes	Lauraceae			1	1					**2**
Baccaurea sp.	Euphorbiaceae									**0**
Platea excelsa	Icacinaceae					1	1			**2**
Campnosperma auriculatum	Anacardiaceae									**0**
Tetramerista glabra	Tetrameristaceae	2	1				1	1	1	**6**
Mangifera gracilipes	Anacardiaceae									**0**
Shorea teysmanniana	Dipterocarpaceae				1		3			**4**
Gymnacranthera contracta	Myristicaceae			1						**1**
Sandoricum beccarianum	Meliaceae			2	2	1			1	**6**
Gluta renghas	Anacardiaceae		2	2	1	2	3			**10**
Parastemon urophyllus	Chrysobalanaceae	2	1	2		4			3	**12**
Dialium patens	Fabaceae				2	1		1		**4**
Brackenridgea palustris	Ochnaceae				2					**2**
Sterculia oblongata	Sterculiaceae		1	2				1		**4**
Garcinia havilandii	Clusiaceae			1	1			1		**3**
Horsfieldia polyspherula	Myristicaceae	1					1	1	1	**4**
**Total**		**6**	**6**	**14**	**11**	**14**	**12**	**6**	**7**	**76**

(c)

**Mothers**													
**Day Nest Tree Species**		**Alice**	**Cissy**	**Dodi**	**Ellie**	**Friska**	**Karma**	**Lilly**	**Lisa**	**Raffi**	**Sarabi**	**Tiara**	**Total**
Dillenia pulchella	Dilleniaceae				3	1							**4**
Alstonia pneumatophora	Apocynaceae					1			1				**2**
Jackiopsis ornata	Rubiaceae	1	4		5	3			3				**16**
Syzygium densiflora	Myrtaceae				11	1			15	1			**28**
Syzygium laxiflorum	Myrtaceae					1		1	1				**3**
Xylopia malayana	Annonaceae				1	1			3				**5**
Terminalia foetidissima	Combretaceae					1							**1**
Glochidion rubrum	Euphorbiaceae						2						**2**
Polyalthia glauca	Annonaceae		2			3				1			**6**
Garcinia sp.	Clusiaceae		4		3	4			3		3		**17**
Litsea gracilipes	Lauraceae		1		6	1	1						**9**
Nothaphoebe umbelliflora	Lauraceae				2								**2**
Baccaurea sp.	Euphorbiaceae				6	1							**7**
Platea excelsa	Icacinaceae					3				1			**4**
Campnosperma auriculatum	Anacardiaceae				1						1		**2**
Tetramerista glabra	Tetrameristaceae		1		12	3			9	2	2	2	**31**
Mangifera gracilipes	Anacardiaceae	1	1		6		1		2				**11**
Shorea teysmanniana	Dipterocarpaceae		2		7	2			5	1			**17**
Gymnacranthera contracta	Myristicaceae		3		2	4		1	5	1			**16**
Garcinia celebica	Clusiaceae				1				1				**2**
Sandoricum beccarianum	Meliaceae		1	1	10	6		1	1	2			**22**
Gluta renghas	Anacardiaceae	2	15		49	52	4		37	7	5	1	**172**
Parastemon urophyllus	Chrysobalanaceae		8	1	13	12	2	1	14	2	1	2	**56**
Dialium patens	Fabaceae		2		10	13		1	6	1	3		**36**
Brackenridgea palustris	Ochnaceae				4								**4**
Sterculia oblongata	Sterculiaceae		2		2	1			1				**6**

Gonystylus sp.	Thymelaeaceae		1										**1**
Macaranga hosei	Euphorbiaceae					1							**1**
Garcinia havilandii	Clusiaceae	1	4		3	1			6	1		1	**17**
Horsfieldia polyspherula	Myristicaceae		9	4	18	13		1	9	3		5	**62**
**Total**		**5**	**60**	**6**	**175**	**129**	**10**	**6**	**122**	**23**	**15**	**11**	**562**

Supplementary Table 3. Night nest tree species use. Use of night nest tree species according to age class; (a) dependent immatures, (b) independent immatures and (c) mothers.

(a)

**Dependent immatures**							
**Night Nest Tree Species**	**Family**	**Chindy**	**Frankie**	**Lilly**	**Lois**	**Nuk**	**Total**
Alstonia pneumatophora	Apocynaceae			1			**1**
Jackiopsis ornata	Rubiaceae			1	1		**2**
Syzygium laxiflorum	Myrtaceae					2	**2**
Tetramerista glabra	Tetrameristaceae	1	1				**2**
Shorea teysmanniana	Dipterocarpaceae	3					**3**
Sandoricum beccarianum	Meliaceae			2	1		**3**
Gluta renghas	Anacardiaceae		2				**2**
Parastemon urophyllus	Chrysobalanaceae		1	4			**5**
Dialium patens	Fabaceae			1			**1**
Sterculia oblongata	Sterculiaceae	1			**1**		**2**
Horsfieldia polyspherula	Myristicaceae	2	2		3		**7**
**Total**		**7**	**6**	**9**	**6**	**2**	**30**

b)

**Independent immatures**<br>**Night Nest Tree Species**		**Chindy**	**Cinnamon**	**Diddy**	**Ellie**	**Fredy**	**Lilly**	**Lois**	**Tina**	**Trident**	**Total**
Agathis sp.	Araucariaceae							1			**1**
Jackiopsis ornata	Rubiaceae							2	5	1	**8**
Syzygium densiflora	Myrtaceae					1	1			2	**4**
Garcinia sp.	Clusiaceae							1		1	**2**
Litsea gracilipes	Lauraceae							1			**1**
Platea excelsa	Icacinaceae							1			**1**
Litsea resinosa	Lauraceae					1					**1**
Campnosperma auriculatum	Anacardiaceae								2		**2**
Tetramerista glabra	Tetrameristaceae				1			2	2		**5**
Mangifera gracilipes	Anacardiaceae					2					**2**
Shorea teysmanniana	Dipterocarpaceae		2		3		1	2	1	7	**16**
Gymnacranthera contracta	Myristicaceae										**0**
Sandoricum beccarianum	Meliaceae	1		1	5	1	2	4	7		**21**
Gluta renghas	Anacardiaceae	2		1	2		1	8	1	3	**18**
Parastemon urophyllus	Chrysobalanaceae	1	3	1			6	8	3	1	**23**
Dialium patens	Fabaceae	1		1		1	1	2	2		**8**
Sterculia oblongata	Sterculiaceae				1				1		**2**
Macaranga hosei	Euphorbiaceae						1		2		**3**
Garcinia havilandii	Clusiaceae						1				**1**
Horsfieldia polyspherula	Myristicaceae	3	1	2	7	5	8	7	4	4	**41**
**Total**		**8**	**6**	**6**	**19**	**11**	**22**	**39**	**30**	**19**	**160**

(c)

**Mothers**<br>**Night Nest Tree Species**		**Alice**	**Cissy**	**Dodi**	**Ellie**	**Friska**	**Karma**	**Lilly**	**Lisa**	**Piniata**	**Raffi**	**Sarabi**	**Tiara**	**Total**
Dillenia excelsa	Dilleniaceae					1								**1**
Alstonia pneumatophora	Apocynaceae										1			**1**
Jackiopsis ornata	Rubiaceae		3		5	6		1	5					**20**
Syzygium densiflora	Myrtaceae		2		2	2	1	1	1					**9**
Syzygium laxiflorum	Myrtaceae		2					2	1					**5**
Xylopia malayana	Annonaceae		4		2	4		1	5					**16**
Polyalthia glauca	Annonaceae				1			1	1		1			**4**
Garcinia sp.	Clusiaceae		3			2			5		2	1	1	**14**
Litsea gracilipes	Lauraceae		1		3	2			4	1	2			**13**
Nothaphoebe umbelliflora	Lauraceae				1									**1**
Platea excelsa Bl.	Icacinaceae		2		2	5			1					**10**
Elaeocarpus petiolatus	Elaeocarpaceae		2		1									**3**
Litsea resinosa	Lauraceae				1	2								**3**
Campnosperma auriculatum	Anacardiaceae		2		2		1		1		1			**7**
Tetramerista glabra	Tetrameristaceae		1		5	1			2		1			**10**
Mangifera gracilipes	Anacardiaceae					1								**1**
Shorea teysmanniana	Dipterocarpaceae	1	8	1	17	12		2	19		2	3	3	**68**
Gymnacranthera paniculata	Myristicaceae							1	1					**2**
Gymnacranthera contracta	Myristicaceae								1		1			**2**
Sandoricum beccarianum	Meliaceae		6	2	24	18		1	14		3			**68**
Gluta renghas	Anacardiaceae	4	10	1	27	20		5	27	2	5	4	1	**106**
Madhuca sericea	Sapotaceae								1					**1**
Parastemon urophyllus	Chrysobalanaceae	1	4		9	7	1	1	12		3	1	1	**40**

Dialium patens	Fabaceae		3	2	4	2			7		2			**20**
Brackenridgea palustris	Ochnaceae				1									**1**
Sterculia oblongata	Sterculiaceae	1	1					1	2					**5**
Macaranga hosei	Euphorbiaceae	1	5		7	13		2	2	1	1			**32**
Garcinia havilandii	Clusiaceae		5		2				2					**9**
Horsfieldia polyspherula	Myristicaceae	5	28	1	31	74		4	63		10	7	7	**230**
**Total**		**13**	**92**	**7**	**147**	**172**	**3**	**23**	**177**	**4**	**35**	**16**	**13**	**702**

Supplementary Table 4. The effect of age of the immature on the probability that they were in peering distance (i.e., within 5 meters) when their mothers were building a nest, analysed with a GLMM with a binomial family distribution (n = 4,435 nest-building scans during 860 follow days on 14 different immature individuals). Including model estimates, standard errors (SE), *P*-values (*P*). Significant *P*-values of the predictors are in bold. The dispersion parameter was 0.995 and the ratio of observed to predicted Zeros was 0.875.

**Factor**	**Factor type**	**Estimate**	**SE**	***P***
Intercept	Intercept	11.527	0.849	<0.001
Age	Predictor	-1.717	0.163	**<0.001**
Individual	Random	-	-	-
Individual: Follow	Random	-	-	-
