# Supplementary material for: Observational social learning of “know-how” and “know-what” in wild orangutans: evidence from nest-building skill acquisition
Source: Commun Biol. 2025 Jun 7;8:890. doi: 10.1038/s42003-025-08217-2 (PMC12145437; doi:10.1038/s42003-025-08217-2)
Supplement: Supplementary file 2 — Reporting Summary [file 42003_2025_8217_MOESM2_ESM.pdf]

nature portfolio

Corresponding author(s): Andrea L. Permana
Caroline Schuppli

Last updated by author(s): 09/04/2025

Reporting Summary

Nature Portfolio wishes to improve the reproducibility of the work that we publish. This form provides structure for consistency and transparency in reporting. For further information on Nature Portfolio policies, see our Editorial Policies and the Editorial Policy Checklist.

Statistics

For all statistical analyses, confirm that the following items are present in the figure legend, table legend, main text, or Methods section.

n/a	Confirmed	
☐	☒	The exact sample size (*n*) for each experimental group/condition, given as a discrete number and unit of measurement
☐	☒	A statement on whether measurements were taken from distinct samples or whether the same sample was measured repeatedly
☐	☒	The statistical test(s) used AND whether they are one- or two-sided<br>*Only common tests should be described solely by name; describe more complex techniques in the Methods section.*
☐	☒	A description of all covariates tested
☐	☒	A description of any assumptions or corrections, such as tests of normality and adjustment for multiple comparisons
☐	☒	A full description of the statistical parameters including central tendency (e.g. means) or other basic estimates (e.g. regression coefficient) AND variation (e.g. standard deviation) or associated estimates of uncertainty (e.g. confidence intervals)
☐	☒	For null hypothesis testing, the test statistic (e.g. *F*, *t*, *r*) with confidence intervals, effect sizes, degrees of freedom and *P* value noted<br>*Give P values as exact values whenever suitable.*
☒	☐	For Bayesian analysis, information on the choice of priors and Markov chain Monte Carlo settings
☐	☒	For hierarchical and complex designs, identification of the appropriate level for tests and full reporting of outcomes
☐	☒	Estimates of effect sizes (e.g. Cohen's *d*, Pearson's *r*), indicating how they were calculated

Our web collection on statistics for biologists contains articles on many of the points above.

Software and code

Policy information about availability of computer code

Data collection	No software was used in data collection.
Data analysis	All analyses and plots were done using R version 4.1.1. Data were analysed using Generalised Linear Mixed Models (GLMM's) as implemented in the lme4 package. For all GLMM models, to assess the overall effect of our predictors, we tested the full model (including all predictors, controls, and random effects) against the null model (including the controls and random effects only) with a likelihood ratio test (LRT) via the anova function. We retrieved the significance of the individual predictors from the model output of the final models. We investigated differences between the multi-level categorical variables using post hoc tests as implemented in the glht function of the multcomp package. For all GLMMs, the DHARMa package in R was used to test for over- / under dispersion and zero inflation using the testDispersion and testZeroInflation functions.

For manuscripts utilizing custom algorithms or software that are central to the research but not yet described in published literature, software must be made available to editors and reviewers. We strongly encourage code deposition in a community repository (e.g. GitHub). See the Nature Portfolio guidelines for submitting code & software for further information.

Data

Policy information about availability of data

All manuscripts must include a data availability statement. This statement should provide the following information, where applicable:

- Accession codes, unique identifiers, or web links for publicly available datasets
- A description of any restrictions on data availability
- For clinical datasets or third party data, please ensure that the statement adheres to our policy

All data needed to reproduce the results presented in this paper are available on GitHub. https://doi.org/10.5281/zenodo.14038374 (2024).

Research involving human participants, their data, or biological material

Policy information about studies with human participants or human data. See also policy information about sex, gender (identity/presentation), and sexual orientation and race, ethnicity and racism.

Reporting on sex and gender	NA
Reporting on race, ethnicity, or other socially relevant groupings	NA
Population characteristics	NA
Recruitment	NA
Ethics oversight	NA

Note that full information on the approval of the study protocol must also be provided in the manuscript.

Field-specific reporting

Please select the one below that is the best fit for your research. If you are not sure, read the appropriate sections before making your selection.

☐ Life sciences ☐ Behavioural & social sciences ☒ Ecological, evolutionary & environmental sciences

For a reference copy of the document with all sections, see nature.com/documents/nr-reporting-summary-flat.pdf

Ecological, evolutionary & environmental sciences study design

All studies must disclose on these points even when the disclosure is negative.

Study description	Standardized activity data recorded at 2-minute intervals were collected by 59 trained researchers and field assistants during focal animal follows via instantaneous sampling. In addition to standard behavioural data, all-occurrence behaviour was recorded during any nest building, nest peering and nest practice behaviour event.
Research sample	Our full data set included 44 recognized individuals of Sumatran orangutans (Pongo abelii): 13 mothers (one of which was followed from independence and one from dependence) and 27 immatures. Of these, 19 were followed only as dependent immatures and 6 individuals followed only as independent immatures. Our data also included 6 individuals followed from dependence through to independence, 1 individual followed as an independent and as a mother and 1 individual followed from dependence throughout independence to motherhood. Immature animals were classed as dependent (constantly travelling with their mother: 0 to around 8 years) or independent (observed at least once without their mother for a minimum of 3 consecutive days but not yet at reproductive age: around 8–15.5 years).
Sampling strategy	Focal animals were followed opportunistically upon encountering them in the forest and for a maximum of 10 consecutive days, after which another focal animal was sought. Consequently, our data often have gaps of several months where individuals were not seen. In addition to activity data, we collected all-occurrence data on nest building, peering events and nest practice behaviour. For each such event, details including the nest-building duration, nest type (day nest or night nest) and nest tree species were recorded on standardized data sheets. This included whether the nest was made in a single or using multiple trees and whether the nest-builder made a lining, pillow, blanket, or roof (collectively referred to as additional comfort elements) whether twigs of the nest were manipulated with the mouth before incorporating them (twig adjustments) and if nest sounds were made during the construction process. All-occurrence data on nest practice behaviour were collected by a subset of the observers and therefore, sample sizes vary between our different analyses.
Data collection	Data were collected by 59 experienced observers on wild Sumatran orangutans at Suaq Balimbing (3° 02.873′ N, 97° 25.013′E) between 2007 and 2024. Data were recorded on tablets or paper data sheets.

Timing and spatial scale	Data were collected during focal animal follows between 2007 - 2024. Focal animals were followed opportunistically upon encountering them in their home range.
Data exclusions	No data points were excluded from the analyses.
Reproducibility	Not applicable - data were collected during direct full day observations of wild animals.
Randomization	Not applicable - data were collected on wild animals without any interactions/ treatments of them.
Blinding	Not applicable - data were collected by observing wild animals in their natural habitats. The animals were individually known to the observers.

Did the study involve field work? ☒ Yes ☐ No

Field work, collection and transport

Field conditions	Work was conducted at the Suaq Balimbing Monitoring Station in South Aceh, Sumatra, Indonesia. The area is a deep peat swamp forest with year-round standing water which can reach chest height in the centre of the study area. The area experiences an everwet climate with two distinct 'wetter' seasons per year. Daily temperatures range from 19-38 degrees celsius.
Location	Suaq Balimbing Monitoring Station in South Aceh, Sumatra, Indonesia (3° 02.873′ N, 97° 25.013′E) covers a 550ha area, 5m above sea level.
Access & import/export	Access to the field site is by way of the Lembang River, which meanders for 15km upstream from it's mouth at the village of Pasir Lembang by the Indian Ocean. No physical samples were collected during this study.
Disturbance	No disturbance was experienced during the study period.

Reporting for specific materials, systems and methods

We require information from authors about some types of materials, experimental systems and methods used in many studies. Here, indicate whether each material, system or method listed is relevant to your study. If you are not sure if a list item applies to your research, read the appropriate section before selecting a response.

Materials & experimental systems

n/a	Involved in the study
☒	☐ Antibodies
☒	☐ Eukaryotic cell lines
☒	☐ Palaeontology and archaeology
☐	☒ Animals and other organisms
☒	☐ Clinical data
☒	☐ Dual use research of concern
☒	☐ Plants

Methods

n/a	Involved in the study
☒	☐ ChIP-seq
☒	☐ Flow cytometry
☒	☐ MRI-based neuroimaging

Animals and other research organisms

Policy information about studies involving animals; ARRIVE guidelines recommended for reporting animal research, and Sex and Gender in Research

Laboratory animals	NA
Wild animals	The study involved observational data collection on a population of wild Sumatran orangutans (Pongo abelii) which are habituated to human presence since the inception of research in 2007.
Reporting on sex	NA
Field-collected samples	NA
Ethics oversight	Ethical approval for our research was granted by the Indonesian Institute of Science (LIPI), the Indonesian State Ministry for Research and Technology (RISTEKDIKTI), the National Research and Innovation Agency (BRIN), the Directorate General of Natural Resources and Ecosystem Conservation under the Ministry of Environment and Forestry of Indonesia (KSDAE-KLHK) and the Gunung Leuser National Park (TNGL) in Indonesia.

Note that full information on the approval of the study protocol must also be provided in the manuscript.

Plants

Seed stocks	NA
Novel plant genotypes	NA
Authentication	NA
